# Idiopathic Non-Dental Facial Pain Syndromes in Italian Children: A Clinical Case Series

**DOI:** 10.3390/life13040861

**Published:** 2023-03-23

**Authors:** Edvige Correnti, Salvatore Lo Cascio, Federica Cernigliaro, Roberta Rossi, Daniela D’Agnano, Giulia Grasso, Annamaria Pellegrino, Barbara Lauria, Andrea Santangelo, Giuseppe Santangelo, Gabriele Tripi, Antonella Versace, Vittorio Sciruicchio, Vincenzo Raieli

**Affiliations:** 1 Child Neuropsychiatry Department, ISMEP, ARNAS Civico, 90100 Palermo, Italy; 2 Child Neuropsychiatry Unit Department, Pro.M.I.S.E. “G. D’Alessandro”, University of Palermo, 90100 Palermo, Italy; 3 Pediatric Headache Center, Pediatric Emergency Department, Regina Margherita Children’s Hospital, 10126 Turin, Italy; 4 Children Epilepsy and EEG Center, San Paolo Hospital, ASL Bari, 70132 Bari, Italy; 5 Pediatrics Department, AOUP Santa Chiara Hospital, 56126 Pisa, Italy

**Keywords:** orofacial pain, children, headache, red ear syndrome, migraine, trigeminal autonomic syndromes

## Abstract

Background. The orofacial pain syndromes (OFPs) are a heterogeneous group of syndromes characterized by painful attacks involving the orofacial structures. They may be summarily subdivided into two great categories: (1) orofacial pain mainly attributed to dental disorders such as dentoalveolar and myofascial orofacial pain or temporomandibular joint (TM) pain; (2) orofacial pain mainly attributed to non-dental pain as neuralgias, facial localization of primary headaches or idiopathic orofacial pain. The second group is uncommon, often described by single case reports, can often show overlapping symptoms with the first group, and represents a clinical challenge, carrying the risk of undervaluation and possibly invasive odontoiatric treatment. We aimed to describe a clinical pediatric series of non-dental orofacial pain and better to underline some topographic and clinical features associated with them. We retrospectively collected the data of children admitted to our headache centers (Bari, Palermo, Torino) from 2017 to 2021. Our inclusion criterion was the presence of non-dental orofacial pain following the topographic criteria of 3° International Classification of Headache Disorders (ICHD-3), and exclusion criteria included the pain syndromes attributed to the dental disorders and pain syndromes due to the secondary etiologies Results. Our sample comprised 43 subjects (23/20 M/F, in the range of ages 5–17). We classified them int: 23 primary headaches involving the facial territory during attacks, 2 facial trigeminal autonomic cephalalgias, 1 facial primary stabbing headache, 1 facial linear headache, 6 trochlear migraines, 1 orbital migraine 3 red ear syndrome and 6 atypical facial pain. All patients described debilitating pain for intensity (moderate/severe), 31 children had episodic attacks, and 12 had continuous pain. Almost all received drugs for acute treatment (less than 50% were satisfied), and some received non-pharmacological treatment associated with drug therapy Conclusion. Although rare OFP can occur in pediatric age, it can be debilitating if unrecognized and untreated, affecting the psychophysical well-being of young patients. We highlight the specific characteristics of the disorder for a more correct and earlier identification during the diagnostic process, already difficult in pediatric age, and to define the approach and possible treatment to prevent negative outcomes in adulthood.

## 1. Introduction

Orofacial pain syndromes (OFPs) are different clinical conditions characterized by pain affecting the orofacial structures, including hard and soft tissues of the head, face and neck [1]. Orofacial pain is defined by the International Classification of Headache Disorders (ICHD) [2], and it primarily consists of musculoskeletal, neurovascular, and neuropathic pain [3,4]. According to the International Classification of Orofacial Pain (ICOP) [5], OFPs are divided into six groups: OFP attributed to disorders of dentoalveolar complex and anatomically related structures to myofascial orofacial pain; temporomandibular joint (TMJ) pain; OFP secondary to lesions or diseases of the cranial nerves; OFP resembling presentations of primary headaches and idiopathic OFP. Treatment can be complex, requiring multiple pharmacological, non-pharmacological, and invasive therapies [3,4]. Furthermore, in the adult population, there is evidence of a high degree of patient dissatisfaction with both the response to therapy and the relationship with the specialists. [5,6]. We also underline that, for more common disorders, the scientific community is making many efforts to develop even more defined and specific criteria and diagnostic modalities for the developmental age, such as those for temporomandibular disorders [7].

With regard to the second group, the criteria are the same as those for adults (both ICDH-3 [2] and ICOP [4]), probably due to the difficulty of bringing together multidisciplinary knowledge. Moreover, distinguishing these disorders is not always easy, considering the possibility of overlapping clinical manifestations and pathophysiological mechanisms [8] and common comorbidities such as sleep disorders in migraine and temporomandibular disorders [9,10,11]. Finally, a further question may arise in this pandemic period regarding COVID-19, asking whether it has led to an increased prevalence and worsening of orofacial symptomatology. Supporting this hypothesis is the observation of greater psychosocial distress in subjects with orofacial pain during this pandemic; however, the data refer only to adults with pain from odontogenic causes [12]. The first three groups almost always involve the odontoiatric and ORL specialists. However, the last three groups (especially V and VI) are often misdiagnosed, and patients undergo invasive treatments. Pediatric OFPs are uncommon but often characterized by high clinical suspicion due to possible symptomatic forms. Therefore, OFPs represent an intriguing clinical challenge, needing a correct diagnosis to recognize secondary forms (like vascular malformations and tumors) and establish a suitable early treatment. Sometimes clinical features are unclear; therefore, the clinician’s approach needs to be careful and supported by neuroimaging [13,14,15,16]. The non-dental orofacial pain is rarely described in childhood [13,14,15]. An important systematic review and meta-analysis of the epidemiological prevalence of primary headaches in childhood have recently been published [17]. Unfortunately, the studies only refer to primary headaches with upper head involvement, and it is impossible to extrapolate the prevalence of migraine and tension-type headaches with facial involvement. To our knowledge, only one study of adults reports data on the prevalence of facial migraines in a population [18]. Hence, we aimed to describe a large clinical pediatric series of these uncommon non-dental primary facial pain to better comprehend such disorders in this age group and avoid a lack of knowledge that may lead to diagnostic errors with possibly dangerous consequences.

## 2. Materials and Methods

The study retrospectively analyzed the clinical records of pediatric outpatients admitted to our centers from 2018 to 2021 (Bari, Palermo, Turin), selecting all children with orofacial pain.

Inclusion Criteria: All selected records reporting the children with localized pain in the upper and lower limits defined by the ICHD-3 [2].

Exclusion Criteria: All records reporting (1) facial pain secondary to the first three groups of the ICOP [4]) or to other secondary causes (demyelinating diseases, tumors, infections, etc.).

We have subdivided and analyzed the children following the syndromic criteria of the ICOP [4]. In Table 1, we list the 6 syndromic groups currently validated by ICOP and the main subgroups.

In addition, we have included all minors with non-dental chronic recurrent orofacial pain in the clinical series even if they had clinical manifestations of as yet unrecognized syndromes, such as red ear syndrome [19,20,21], primary stabbing headache [22,23] or facial linear headache [24,25]. These are disorders that now have extensive literature [26]. On the other hand, the current classification (ICOP) [4] does not report certain disorders, which are validated by the ICHD-3 [2], such as Nervus Intermedius Neuralgia or Neck-Tongue Syndrome [2]. This is because it is proposed, as the Authors say, as an evolving classification and is the first of its kind. To this end, the description of an initial extensive pediatric case history seemed useful and met the aims of the classifiers.

Furthermore, to understand if the pain site could be associated with a specific syndrome, we also subdivided the children following anatomical localization of pain specified into 10 anatomo-topographic pain focus:

- Orbit: the area encompassing the eye and contour region.
- Trochlear: when pain is localized in an innermost region; superomedial orbit, extended to the forehead [5].
- Nasal root: from the middle-frontal-basal region to the first portion of the nose.
- Maxilla: includes maxillary and sub-orbitary region.
- Ear: entire earcup and contour region of cheeks.
- Under ear: the region immediately below to earcup.
- Mandibular: includes mandibular and temporo-mandibular (innermost region compared to the previous) region.
- Mouth.
- Facial: non-specified facial region.
- Variable: localized in two or more areas: ear, periorbital region, dental arch, nose, facial.

The other information that we collected is:

- use of pharmacological analgesic therapy and possible response.
- use of prophylaxis and possible benefit.
- familiarity.

Informed consent was obtained from the parents of all individuals. According to local ethical policies, no formal approval by the hospital ethics committee was needed.

## 3. Results

### 3.1. General Data

Data were collected from 43 patients, with an average age of 10.7 ± 3.41 years. All patients described a history of debilitating pain for intensity (moderate/severe), 31 children had episodic attacks, and 12 had continuous pain.

Using over mentioned criteria, we identified the following painful syndromes:

- 23 primary orofacial pain V group involving the facial topography, with features resembling primary headaches (in this case, we have detached ourselves from the ICOP classification [4], which defines orofacial pains of group V only as those primitively localized on the face and not those cranial pains which also involve the face, see notes to the classification);
- 2 facial TACs;
- 1 facial primary stabbing headache;
- 1 facial linear headache;
- 6 trochlear migraine;
- 1 orbital migraine
- 3 red ear syndrome with irradiation in the anterior facial;
- 6 Persistent idiopathic facial pain (PIFP).

### 3.2. Topographic Distribution of Pain

The distribution of pain in the anatomic area was particularly various.

We observed that TACs were strictly localized at the orbital site. In contrast, migraine with irradiation to the face typically may show a progressively extended distribution (orbital, nasal root, maxilla, mouth, and mandible).

One female patient presented a labial localization of her episodic pain in the contest of migraine.

We defined trochlear migraine pain as strictly localized in the infra-trochlear angle (described previously) [27,28], and we observed six patients with strictly orbital pain (rarely associated with frontal and temporal irradiation). Red ear cases were included in our sample since they usually display large pain irradiation to the cheeks. Idiopathic facial pain always involves the lower half of the face.

**Figure 1:**
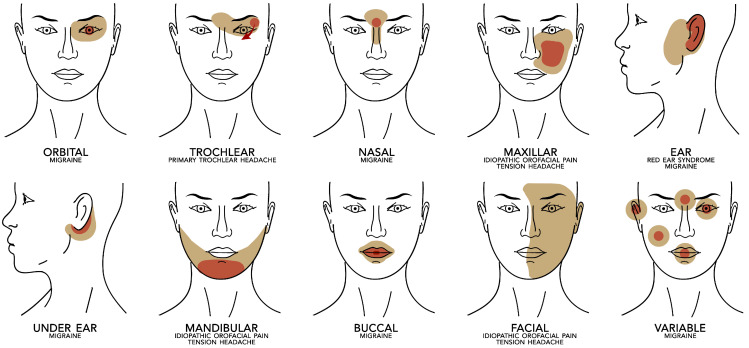
Distribution of pain on the anatomic area with prevalent associated diagnosis. Red represents the focal point of pain presentation, and yellow is the adjacent areas of prevalent irradiation.

We used two sets of categories to enclose the other patients who had a variable localization of pain or who could not describe the precise indication of the pain. Data are summarized in Table 2 and illustrated in Figure 1.

### 3.3. Clinical Syndromes

Our sample has been subdivided into different clinical syndromes following the ICOP criteria. Unsurprisingly, episodic syndromes prevailed, although migraine-like clinical syndromes and idiopathic facial pain showed several subjects with chronic or continuous pain. Regarding the use and response to pharmacological therapies, we observed that, out of the entire sample, 83.7% (36/43) resorted to symptomatic therapy, which yielded a positive response in 41.6% (15/36) of the subjects. In comparison, only 51.6% (22/43) of the sample underwent prophylactic therapy, with a very low reported positive response of 13.6% (3/22) of children. We can therefore conclude that the response to analgesic drugs or prophylaxis therapy is low. In Table 3 we summarized the data about clinical syndromes subdivided for age, gender, temporal course, symptomatic and preventive therapy.

### 3.4. Range of Age

Considering that there may be variations in the clinical phenotype during developmental age and certain syndromes may appear at different ages [29,30], we also analyzed our sample according to age group. We divide the population into three ranges of age, 5–8, 9–13 e 14–17. We obtain this distribution explicitly in the graphic in Table 4.

## 4. Discussion

The orofacial region comprises several structures and tissues, including the facial skin, oral mucosa, teeth and surrounding periodontal tissues, periosteum, bone, the temporomandibular joint (TMJ), muscles, ligaments, fascia, etc. [31,32]. The face and mouth perform important functions, including eating, drinking, speech and expression of emotions. Furthermore, they have a large somatosensory representation in the cortex. For these reasons, this region is also the site of some of the most common acute and chronic pain conditions. It should not be surprising, given that their involvement in nociceptive processes and providing painful sensations impacts important emotional and behavioural disturbances, making their treatment difficult since it cannot be limited to analgesic therapy alone. [31]. Suggestions from experimental and clinical studies [31,32] suggest that amplification of the sensitive stimuli in the periphery and/or in the central nervous system can promote painful disorders such as orofacial pain, while reduction of nociceptive inputs may decrease the sensitization provoked by nociceptive inputs to the CNS [3,26,31]. The decrease in central sensitization, as shown by decreased receptive field size, relieves pain and reduces allodynia [32]. The central sensitization in persistent orofacial pain is probably promoted by an imbalance of descending inhibition and facilitation [31]. More descending facilitation can elicit and sustain chronic craniofacial pain, both in the presence of local injury and functional conditions such as migraines. Changes in the descending modulatory system induced by the hypothalamus, periaqueductal grey matter, or rostro ventralmedial medulla may influence pain control. Several painful orofacial conditions (V neuropathic pain, migraine, TMJ, and TACs) may be linked to these functional/lesional changes that can have a predictive role in developing chronic orofacial pain [31,32]. In the development of chronic orofacial pain conditions, an important role is certainly played by inflammatory processes. The presence of multiple structures that make up this region facilitates the possibility of inflammatory processes that can initiate that process of peripheral sensitization. These inflammatory processes involve several mediators released from mast cells, immune cells, macrophages, and injured cells acting on ion channels or membrane receptors localized in the nociceptive afferent nerve endings and thereby may start peripheral sensitization [33]. What has been briefly mentioned above from the point of view of pathophysiological mechanisms gives a good idea of the complexity of factors to be evaluated in the approach to orofacial pain [16]. In addition, the difficulty of conducting experimental studies in developmental age makes it difficult to draw specific conclusions solely from experimental animal models or performed in adults.

If untreated, non-dental facial pain is a debilitating disorder that is often not recognized or attributed to odontogenic or myofascial causes in adults and children [13,14,15,16]. The recently published ICOP classification [4] sought to establish diagnostic criteria to better define the different subgroups to validate them with further research. However, facial neuralgias are otherwise rare in children. An example of the confusion in defining the characteristics of orofacial pain in children is enlightened in a recent review [34]. The scientific literature on the last three ICOP groups refers to the typical migraine and tensive forms affecting the head. Even the interesting review by Grazzi et al. (2018) [14] has difficulty reporting data, especially relating to the V and VI groups of the ICOP classification. A possible explanation for this lack of data may lie in the rarity of these disorders in childhood and adolescence and the likely lack of recognition of painful facial forms, which will usually be sent to dentists or otolaryngologists, and hardly to neuro-pediatricians [5,6]. This suggestion is supported by observations in adults in selected clinical specimens [16], which show that patients with primary headaches or neuralgia often undergo dental examinations and invasive dental procedures. In addition, we have recently found in a general population sample those patients with non-dental orofacial pain received several diagnoses and frequently underwent dental treatment [6].

To our knowledge, our study presents the first large population of pediatric patients affected by non-dental orofacial pain. In our sample, more than half of the children reported cranial migraine attacks involving the lower part of the head. While the ICOP [4] strictly classifies them as migraines and not as belonging to the V ICOP Group, we chose to include them in our study as they complained of severe pain in this area. Several children underwent dental examinations for this atypical irradiation, despite the episodic and throbbing characteristics of the pain associated with typical neuro vegetative signs.

In the only study on orofacial involvement in adult migraine [18], about 10% of the subjects had migraine attacks involving the face, similar to our sample, while only 0.2% had only facial location of migraine. Very interesting was the datum that migraineurs with orofacial irradiation had more cranial autonomic symptoms (CAS) than migraineurs without orofacial involvement [35]. The presence of CAS in migraine, similarly to the TACs, is an object of much scientific interest [36] for probable different childhood and adult prognosis [37] and answer to the treatment in these subgroups of migraineurs. Also, adult migraineurs are often first seen by their dentist, and the underlying migraine symptoms remain unrecognized or are frequently misdiagnosed as trigeminal neuralgia or “sinus headache” [36]. In our sample, one characteristic site is the orbital site, which all our CT cases reported and also is the chosen site of a particular picture of Migraine, called “Trochlear”. Trochlear migraine was recently described by us Raieli 2020 [27,28], suggesting a modification of the definition given by the Spanish researchers [38,39].

This disorder fulfils all the diagnostic criteria for migraines without aura, but it is strictly localized to the infra-trochlear site and is painful even when the eyes move. It should be distinguished from inflammatory trochleitis [40]. In our sample, we also report the case of a child who almost always had attacks in the right orbital site, pulsating, with rare irradiation in the frontotemporal site, unresponsive to preventive therapy. To make the correct diagnosis, the clinician has to distinguish facial pain from headache, defined by ICHD-3 [2] as “pain located in the head or the face above the orbitomeatal line”. This arbitrary line corresponds to the border between the ophthalmic (V1) and maxillary (V2) branches of the trigeminal nerve, connecting the external auditory meatus and the outer canthus. The definition of OFP also includes the orbit, considering headaches as OFPs, suggesting that the orbit should be excluded from this definition [34]. Recently Ziegler and May (co-authors of the ICOP classification) [10] criticize the topographic criteria established by the IHS to define pain as orofacial. In particular, the upper limit of the orofacial territory given by the orbito-meatal line, involving the orbit, would make many cases of TACs and also of migraines as orofacial pain of ICOP group V [4]. This overlap with the classical pictures of TACs of the 3rd ICHD could not allow the evaluation of possible clinical-therapeutic differences between disorders belonging to groups 1 and 3 of the 3rd ICHD and ICOP group V. They say, “The ICHD’s current definition of facial pain, including the eyes, renders this definition unprecise. The issue becomes important or indeed decisive when facial pain has to be distinguished from headache syndromes”. Therefore, they propose to move the upper limit for defining pain as orofacial to the infra-orbitomeatal line (IOML) connecting the Porus acousticus externus and the Margo infraorbitalis or the acanthio-meatal line (AML) connecting the Porus acousticus externus and the acanthion [41]. Surely, an important issue has to be discussed in the coming years to understand the real differences between primary headaches and orofacial pain resembling primary headaches (ICOP group V) [4].

Continuing the discussion in relation to the topography of orofacial pain in the group mainly involving the lower half of the face (suborbital), our sample reports 6 subjects meeting the diagnostic criteria of persistent idiopathic facial pain (ICOP group VI) and some subjects presenting with pictures not yet recognized by current classifications but of which there is literature such as linear headache [24,25] and in the case of red ear syndrome, particularly numerous [19], especially in the pediatric population [15,19]). The rarity or absence in our sample of cases of tension headache or neuralgia is not surprising because in the first case, in addition to considering the atypical and often misdiagnosed site, they are infrequent in clinical populations due to the lesser severity of the attacks, which hardly leads to a specialist evaluation, while in the second case, the neuralgias are almost always secondary, as well as being very rare in the pediatric age [26].

This study provides insights into the symptomatic and prophylactic treatment of non-dental orofacial pain. However, managing this condition is challenging due to comorbidities such as sleep disturbances, stress, and psychological disorders [3,31]. To our knowledge, no studies on treating paediatric non-dental orofacial pain exist. Therefore the symptomatic and prophylactic treatment strategies generally in individual cases reported following the criteria used in primary headaches with cranial involvement. Prophylactic therapy is not commonly used in pediatric non-dental orofacial pain, and the limited number of cases [42] does not allow for significant conclusions. However, our sample generally shows a low response to the treatment. Less surprise arises about the low response to the treatment in persistent idiopathic facial pain, similar to the adult population [16]. These findings suggest that non-dental orofacial pain is often refractory to the therapy and requires a multidisciplinary approach, possibly avoiding invasive dental treatments.

Furthermore, the retrospective nature and the highly variable follow-up did not allow comparisons between treated and untreated subjects. The data in Table 3 seem suggestive, showing how using analgesics and prophylactic therapies increases with age, while the response decreases. The forms of idiopathic persistent orofacial pain mainly affect adolescents. However, studies with larger populations are needed to confirm these data.

Finally, our study certainly has the limitations of a retrospective study, with a heterogeneous sample, partly operator dependent on data collection. However, in addition to having collected data from three centers specialized in the treatment of headaches, it is the first study to our knowledge that draws attention to this group of pediatric patients. Furthermore, although large in its total, the sample did not allow useful comparisons between the different syndromes because the numerical consistency of each was heterogeneous. Moreover, given the retrospective nature of our work, although some data were reported, we preferred not to describe the short and long-term follow-up because of the heterogeneity of the syndromes presented and the small number of some of them. For these reasons, it is impossible to give specific indications of the long-term development of these disorders compared with what is known concerning primary headaches with cranial involvement.

It seems important to us to focus the attention of pediatricians and neuropsychiatrists on this topic since, especially in the high-frequency or chronic forms, the OFPs may be disabling conditions, often characterized by the presence of comorbidities, such as psychological and behavioral factors, sleep disorders, headache, systemic disorders, and trauma. Therefore, the psychosocial assessment of these patients plays a very important role in the multidisciplinary approach to OFPs: psychological counseling, relaxation training, biofeedback, CBT, physical self-regulation, and mindfulness-based interventions can decrease the intensity and frequency of the pain, improving the quality of life and preventing disability. Lifestyle is also crucial, with regular and healthy nutrition, avoiding triggering factors like an overloaded daily routine. Finally, in some cases, pharmacotherapy may be required [42].

## 5. Conclusions

OFP is a debilitating disorder, although rare in pediatric age. Failure recognition of this condition can lead to erroneous interventions and, over time, affect the psychophysical well-being of young patients. With our study, the first Italian study on a large pediatric OFP series, we aimed to underline the specific characteristics of the disorder with the purpose of a more correct and earlier identification during the diagnostic process, which is already difficult in pediatric age, and to define an approach and possible treatment to prevent negative outcomes in adulthood.

## Figures and Tables

**Table 1:** 1°st International Classification of Orofacial Pain (ICOP) 2020 [4].

Diagnosis	ICOP Subgroups
1. Orofacial pain attributed to disorders of dentoalveolar and anatomically related structures	1.1 Dental pain 1.1.1 Pulpal pain 1.1.2 Periodontal pain 1.1.3 Gengival pain 1.2 Oral mucosal, salivary gland and jawbone pains 1.2.1 Oral mucosal pain 1.2.2 Salivary gland pain 1.2.3 Jaw bone pain
2. Myofascial orofacial pain	2.1 Primary myofascial orofacial pain 2.1.1 Acute primary myofascial orofacial pain 2.1.2 Chronic primary myofascial orofacial pain 2.2 Secondary myofascial orofacial pain 2.2.1 Myofascial orofacial pain attributed to tendonitis 2.2.2 Myofascial orofacial pain attributed to myositis 2.2.3 Myofascial orofacial pain attributed to muscle spasm
3. Temporomandibular joint (TMJ) pain	3.1 Primary temporomandibular joint pain 3.1.1 Acute primary temporomandibular joint pain 3.1.2 Chronic primary temporomandibular joint pain 3.2 Secondary temporomandibular joint pain 3.2.1 Temporomandibular joint pain attributed to arthritis 3.2.2 Temporomandibular joint pain attributed to disc displacement 3.2.3 Temporomandibular joint pain attributed to degenerative joint disease 3.2.4 Temporomandibular joint pain attributed to subluxation
4. Orofacial pain attributed to lesion or disease of the cranial nerves	4.1 Pain attributed to lesion or disease of the trigeminal nerve 4.1.1 Trigeminal neuralgia 4.1.2 Other trigeminal neuropathic pain 4.2 Pain attributed to lesion or disease of the glossopharyngeal nerve 4.2.1 Glossopharyngeal neuralgia 4.2.2 Glossopharyngeal neuropathic pain
5. Orofacial pains resembling presentations of primary headaches	5.1 Orofacial migraine 5.1.1 Episodic orofacial migraine 5.1.2 Chronic orofacial migraine 5.2 Tension-type orofacial pain 5.3 Trigeminal autonomic orofacial pain 5.3.1 Orofacial cluster attacks 5.3.2 Paroxysmal hemifacial pain 5.3.3 Short-lasting unilateral neuralgiform facial pain attacks with cranial autonomic symptoms 5.3.4 Hemifacial continuous pain with autonomic symptoms 5.4 Neurovascular orofacial pain 5.4.1 Short-lasting neurovascular orofacial pain 5.4.2 Long-lasting neurovascular orofacial pain
6. Idiopathic orofacial pain	6.1 Burning mouth syndrome 6.1.1 Burning mouth syndrome without somatosensory changes 6.1.2 Burning mouth syndrome with somatosensory changes 6.1.3 Probable burning mouth syndrome 6.2 Persistent idiopathic facial pain 6.2.1 Persistent idiopathic facial pain without somatosensory changes 6.2.2 Persistent idiopathic facial pain with somatosensory changes 6.2.3 Probable persistent idiopathic facial pain 6.3 Persistent idiopathic dentoalveolar pain 6.3.1 Persistent idiopathic dentoalveolar pain without somatosensory changes 6.3.2 Persistent idiopathic dentoalveolar pain with somatosensory changes 6.3.3 Probable persistent idiopathic dentoalveolar pain 6.4 Constant unilateral facial pain with additional attacks

**Table 2:** Demographic and clinical characteristics of the 43 patients included in the follow-up study.

**Area**	**Unit (n°)**	**Mean Age (y)**	**Frequence (%)**	**Diagnosis**
Orbit	7	8.6	Episodic: 75 Chronic: 25	Migraine orbital migraine TACs
Trochlear	6	10.4	Episodic: 100 Chronic: 0	Trochlear Migraine
Nasal Root	5	9.6	Episodic: 60 Chronic: 40	Migraine
Maxilla *	2	11	Episodic: 50 Chronic: 50	PIFP Migraine
Ear	4	11	Episodic: 75 Chronic: 25	Migraine RES
Under Ear	1	10	Episodic	Migraine
Mandible °	4	12.2	Episodic: 75 Chronic: 25	PIFP Migraine linear headache
Mouth	1	16	Episodic	Migraine
Facial #	4	12.75	Episodic: 0 Chronic: 100	PIFP
Variable +	9	10.5	Episodic: 75 Chronic: 25	Migraine Tension Headache

* Includes maxillary and sub-orbitary pain. ° Includes mandibular and temporo-mandibular pain. # Not specificated facial region. + Localized pain in two o more areas: ear, peri-orbitary region, dental arch, nose, facial. TACs. trigeminal autonomic Cephalalgias syndromes. PIFP: Persistent idiopathic facial pain. RES: Red Ear Syndrome.

**Table 3:** Date analyzed based on the diagnosis.

Diagnosis	Unit (n°)	Mean Age (y)	Sex (n)	Frequence (%)	Use of Analgesics (%)	Analgesics Response (%)	Prophylaxis (%)	Prophylaxis Benefit (%)
Migraine	17	10.6	M: 8 F: 5	Episodic: 70 Chronic: 30	84	45	61	10
Trochlear Migraine	6	10.1	M: 5 F: 1	Episodic: 100 Chronic: 0	100	66	0	-
Tension Type	7	11.4	M: 0 F: 7	Episodic: 72 Chronic: 28	71	40	42	33
Trigeminal Autonomic	2	5	M: 2 F: 0	Episodic: 100 Chronic: 0	50	100	0	-
Red Ear Syndrome	3	6.5	M: 2 F: 1	Episodic: 50 Chronic: 50	100	33	66	0
stabbing H. headache facial linear headache	2	10.3	M: 1 F: 1	Episodic: 1 Chronic: 1	100	0	50	0
Persistent idiopathic facial pain	6	12.3	M: 2 F: 4	Episodic: 50 Chronic: 50	83	20	100	16

Legend: M: male. F: female. The percentage of Analgesics Response is the number of patients who used analgesics. The percentage of Prophylaxis Benefit is considered to the number of patients who did prophylaxis.

**Table 4:** Date analyzed with division into age ranges.

DATA	Range 5–8 y	Range 9–13 y	Range 14–17 y
Unit (n°)	11	23	9
Sex	M: 6 F: 5	M: 15 F: 8	M: 1 F: 8
Mean age (y)	6.3	11	15.4
Frequence (%)	Episodic: 81.9 Chronic: 18.1	Episodic: 73 Chronic: 27	Episodic: 55 Chronic: 45
Prevalent Area	Orbit	Trochlear/nasal/maxill	Facial/Variable ^+^
Prevalent Diagnosis	Migraine	Migraine	PIFP/tension headache
Use of Analgesics (%)	73	86	89
Analgesics Responce (%)	50	47	25
Prophylaxis (%)	45	40	78
Prophylaxis Benefit (%)	20	11	14

The percentage of Analgesics Response is referred to the number of patients who used analgesics. The percentage of Prophylaxis Benefit is considered to the number of patients who did prophylaxis. + Localized pain in two or more areas: ear, peri-orbitary region, dental arch, nose, and facial. PIFP: Persistent idiopathic facial pain.

## Data Availability

No new data were created, and clinical data are unavailable due to privacy or ethical restrictions.
